# Regulation of Cytokines and Dihydrotestosterone Production in Human Hair Follicle Papilla Cells by Supercritical Extraction-Residues Extract of *Ulmus davidiana*

**DOI:** 10.3390/molecules27041419

**Published:** 2022-02-19

**Authors:** Ye-Eun Kwon, Sun-Eun Choi, Kwang-Hyun Park

**Affiliations:** 1 Department of Forest Biomaterials Engineering, College of Forest & Environmental Sciences, Kangwon National University, Chuncheon 24341, Korea; kye0519@naver.com; 2 Department of Emergency Medicine and BioMedical Science Graduate Program (BMSGP), Chonnam National University, Gwangju 61469, Korea; 3 Department of Emergency Medical Rescue, Nambu University, Gwangju 62271, Korea

**Keywords:** *Ulmus davidiana* extract, residue of supercritical fluid extraction, H_2_O_2_-induced cytokine secretion, dihydrotestosterone, catechin-glycoside, human dermal follicle papilla cells (HDFPCs)

## Abstract

This study was conducted to examine the anti-hair loss mechanism of the supercritical fluid extraction-residues extract of *Ulmus davidiana* by the regulation of cytokine production and hormone function in human dermal follicle papilla cells (HDFPCs). To investigate the modulatory effects on H_2_O_2_-induced cytokines, we measured transforming growth factor-beta and insulin-like growth factor 1 secreted from HDFPCs. To investigate the regulatory effects of supercritical extraction-residues extract of *Ulmus davidiana* on dihydrotestosterone hormone production, cells were co-incubated with high concentrations of testosterone. The supercritical extraction-residues extract of *Ulmus davidiana* significantly inhibited the secretion of transforming growth factor-beta but rescued insulin-like growth factor 1 in a dose-dependent manner. The supercritical extraction-residues extract of *Ulmus davidiana* markedly reduced dihydrotestosterone production. These results suggest that the supercritical fluid extract residues of *Ulmus davidiana* and their functional molecules are candidates for preventing human hair loss.

## 1. Introduction

*Ulmus davidiana* (UD), a well-known traditional medicine in Korea and worldwide, has been used to treat various diseases [1]. Previous medicinal purpose-based experiments reported that UD had anti-oxidative, anti-inflammatory, anti-tumorogenic, and neuroprotective effects [2,3].

Recently, UD has also been used as an edible food-based material to provide amino acids [4], oligosaccharides [5], and other unidentified functional metabolites and is used in many biochemical metabolic processes.

Many reports confirmed that UD contains a number of useful molecules [4,6], the precise components of which vary depending upon the isolation method [5,7]. Its functionality has been studied in Asia, Europe, North America, and Korea [8], but its function in hair health and the prevention of hair loss is unclear. 

Previous research indicated that UD extracts had various ameliorative effects on acute inflammatory responses in rats [9,10], osteopenia [11], and an in vitro model [12]. Similarly, the supercritical fluid of UD had anti-inflammatory, anti-angiogenesis [13], and anti-melanin effects [14].

At the initiation of alopecia, dihydrotestosterone (DHT) synthesis is increased by transforming growth factor-β (TGF-β) in dermal papilla cells [15]. Then, TGF-β antagonists were suggested as effective candidates for promoting the elongation of hair follicles. Furthermore, the synthetic mechanism of TGF-β in dermal papilla cells was closely correlated with the activation of the intrinsic caspase network for programmed cell death. Therefore, these sequential pathways are involved in regulating the dermal hair cycle.

IGF-1 was reported to be a member of reciprocal signaling and is expressed in the dermal cells. Previous reports were confirmed by the lack of response of androgenetic-type alopecia to treatment with topical minoxidil and the low effects of oral finasteride in pituitary gland-resected patients [16]. Moreover, the effects of IGF-1 provide further evidence for promoting hair growth and treating alopecia, but a correlative pathway between IGF-1, TGF-β, and DHT has not been reported.

Therefore, this study was performed to investigate the effects of the supercritical fluid extraction-residues extract of UD including molecules such as phenolic compounds, hydrophilic substances, and flavonoids on oxidative stress-induced cytokine secretion and the recovery of hormone regulation function in an in vitro model. 

## 2. Results and Discussion

**Figure 1:**
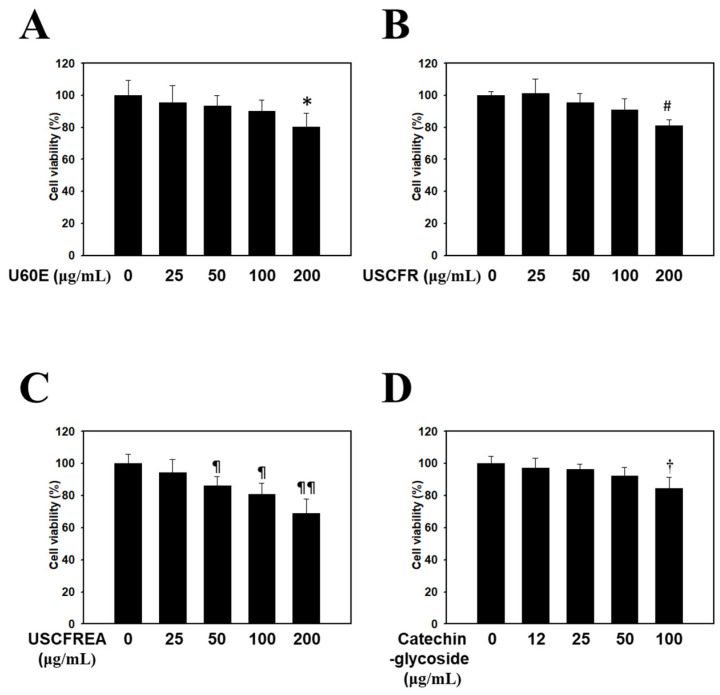
Endogenous cytotoxicity of *Ulmus* extract fractions and catechin-glycoside in HDFPCs. Cells were incubated with the indicated doses of (**A**) U60E, (**B**) USCFR, (**C**) USCFREA, and (**D**) catechin-glycoside (μg/mL, *w*/*v*). Cell viability was calculated as described in Materials and Methods. Data are expressed as the mean ± SD. * *p* < 0.05, ^#^ *p* < 0.05, ^¶^ *p* < 0.05, ^¶¶^ *p* < 0.01, and ^†^ *p* < 0.05 vs. the untreated group.

HDFPCs were treated with various concentrations of *Ulmus* extracts and catechin-glycoside for 12 h. Cell viability was determined by the MTT assay. The cytotoxicity of the *Ulmus* extracts and catechin-glycoside is presented in Figure 1. The cytotoxicity of U60E (Figure 1A), USCFR (Figure 1B), USCFREA (Figure 1C) and catechin-glycoside (Figure 1D) (μg/mL, *w*/*v*) was higher than 80% at nearly 100 μg/mL. The *Ulmus* extracts and catechin-glycoside results showed favorable safe properties at relatively high concentrations.

The administration of TGF-β1 to experimental in vivo models initiated the proliferation of hair follicle-derived papilla cells and reduced the number of keratinocytes. Furthermore, TGF-β receptor II agonists and/or antagonists were suggested as useful therapeutic agents to regulate the expression of TGF-β1 for human-hair-related disorders based on hirsutism and/or delayed/early degenerative alopecia [17]. Therefore, these reports suggest that the amount of TGF-β1 released from hair papilla cells is closely correlated with androgen-dependent alopecia.

In general, reactive oxygen species (ROS) formation was increased by androgen treatment, and TGF-β1 secretion was induced by the treatment of the DP-6 cell line with androgen, whereas androgen-induced TGF-β1 secretion was significantly reduced by n-acetyl cysteine (NAC) [18]. Therefore, androgen-induced TGF-β1 signals can be blocked by treatment with antioxidants to interrupt the ROS-mediated signaling pathway in hair follicle cells.

**Figure 2:**
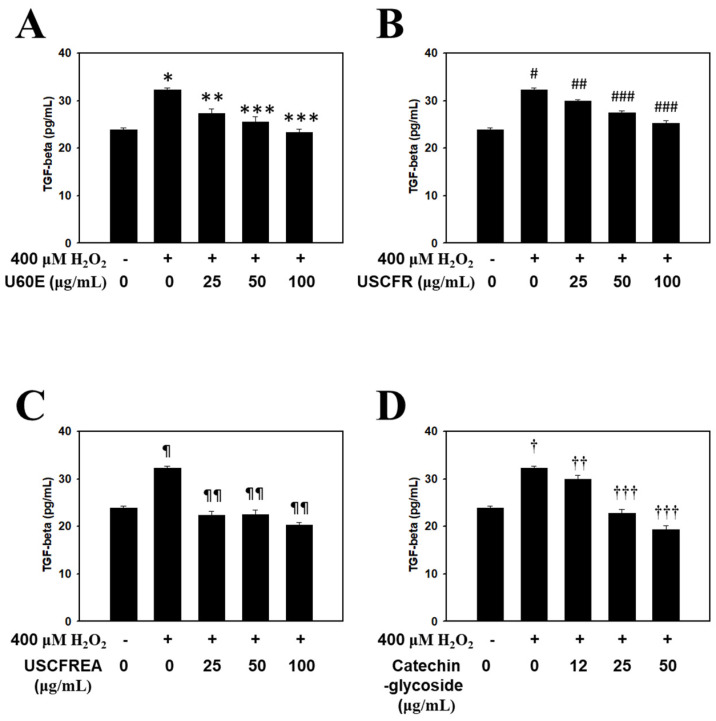
Inhibitory effect of *Ulmus* extract fractions on oxidative−stress−induced TGF−β production. Cells were pre−incubated with the indicated doses of (**A**) U60E, (**B**) USCFR, (**C**) USCFREA, and (**D**) catechin−glycoside (μg/mL, *w*/*v*) and treated with H_2_O_2_ for 12 h. TGF−β production was measured as described in Materials and Methods. Data are expressed as the mean ± SD. * *p* < 0.05, # *p* < 0.05, ^¶^ *p* < 0.05 and ^†^ *p* < 0.05 vs. normal group (blank). ** *p* < 0.05, *** *p* < 0.005, ^##^ *p* < 0.05, ^###^ *p* < 0.01, ^¶¶^ *p* < 0.005, ^††^ *p* < 0.05 and ^†††^ *p* < 0.001 vs. control group (H_2_O_2_ treated only).

**Figure 3:**
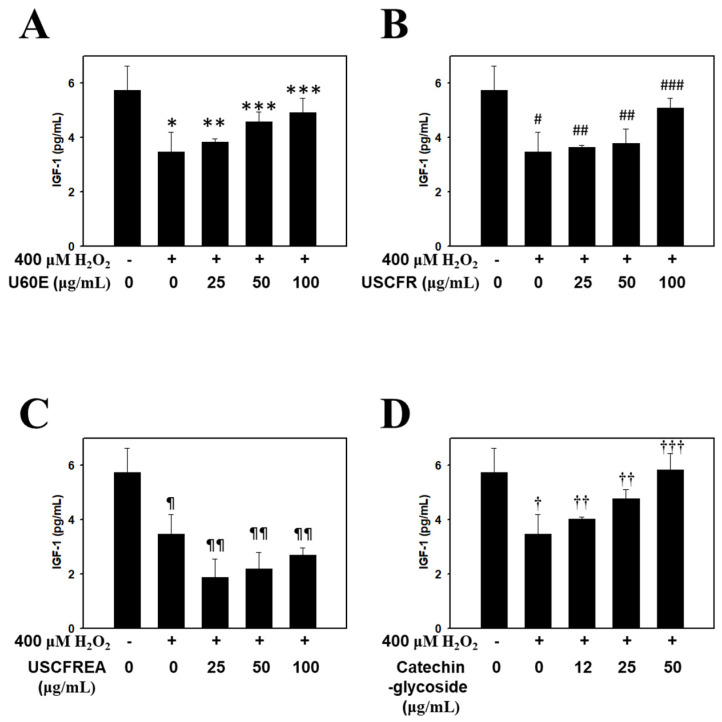
Recovery effects of *Ulmus* extract fractions on oxidative−stress−induced IGF−1 formation. Cells were pre-incubated with the indicated doses of (**A**) U60E, (**B**) USCFR, (**C**) USCFREA, and (**D**) catechin−glycoside (μg/mL, *w*/*v*) and treated with H_2_O_2_ for 12 h. IGF−1 production was measured as described in Materials and Methods. Data are expressed as the mean ± SD. * *p* < 0.05, ^#^ *p* < 0.05, ^¶^ *p* < 0.05 and ^†^ *p* < 0.05 vs. normal group (blank). ** *p* < 0.05, *** *p* < 0.01, ^##^ *p* < 0.05, ^###^ *p* < 0.01, ^¶¶^ *p* < 0.005, ^††^ *p* < 0.05 and ^†††^ *p* < 0.001 vs. control group (H_2_O_2_ treated only).

The present study confirmed that the *Ulmus* extracts and catechin-glycoside inhibited TGF-β1 secretion, which simultaneously accelerated hair loss while inhibiting hair growth. The effect of H_2_O_2_, U60E (Figure 2A), USCFR (Figure 2B), USCFREA (Figure 2C), and catechin-glycoside (Figure 2D) on TGF-β1 secretion is shown in Figure 3. The expression of TGF-β in the H_2_O_2_ group was significantly increased compared to the control group. Interestingly, the parallel administration of H_2_O_2_ and *Ulmus* extracts or catechin-glycoside resulted in a significant decrease in the secretion of TGF-β. Therefore, these results suggest that *Ulmus* extracts and catechin-glycoside are excellent natural new materials that could be used as hair care agents for the prevention of hair loss and/or the stimulation of hair growth.

In hair follicle papilla cells, IGF-1 was reported as the pivotal growth factor involved in the stimulation or regulation of hair growth by promoting the growth of cultured epithelial cells [15,19]. Especially, IGF-1 expression was increased by stimulation from androgenic hormones and testosterone activity in the hair. Therefore, IGF-1 is known to play an important role in the mechanism of action of androgen-dependent alopecia [20,21]. In addition, IGF-1 was involved in preventing aging, preventing adult aging-related diseases, including skin aging, enhancing immunity, improving bone density, alleviating dementia and depression, and inducing the transformation of hair from a resting state to a growing state [22]. Furthermore, hair growth is closely correlated with blood circulation, and IGF-1 and EGF contribute to the growth of hair follicles that promote the growth of hair [23,24]. As such, increasing the expression of IGF-1, which is an important factor in hair growth, is expected to be effective in hair growth. Discovering the advantages of developing new materials derived from natural products that minimize the risk of side effects and the toxicity of existing synthetic-based compounds is important for developing new technologies.

Thus, in the present study, we confirmed the effect of promoting the expression of IGF-1 in a concentration-dependent manner by treating dermal papilla cells with U60E (Figure 3A), USCFR (Figure 3B), USCFREA (Figure 3C), and catechin-glycoside (Figure 3D). The results suggest that the single compound catechin-glycoside and its extract have hair growth-promoting effects, and suggests the possibility of their development as natural new agents for hair growth.

In general, it is known that male pattern hair loss is determined by genetics, and when hair loss begins earlier, the degree of hair loss tends to become more severe. Hair loss symptoms are reported to have a strong genetic association, especially in males, but a previous study reported that if at least one of the paternal or maternal family members had alopecia, then this condition was more likely to specifically develop in males from later generations [25,26].

However, one of the representative factors causing hair to thin or fall out is that testosterone, a male hormone, is changed to dihydrotestosterone (DHT) by 5α-reductase, which causes the shrinkage of the dermal papilla of the scalp. The results suggest that the single-compound catechin-glycoside and its extract have hair growth-promoting effects. DHT is generated, and eventually, hair loss proceeds [27]. Male hormones promote the positive function of masculinity and cause seborrheic dandruff to worsen the symptoms of alopecia [28]. DHT acts on hair cells, causing atrophy and slowing cell division of the hair follicles, resulting in softening of the hair and hair loss [29]. Minoxidil, a compound used in the positive control group in this experiment, was developed as a vasodilator for the treatment of hypertension in the early 1970s but has been used as a hair growth promoter after hirsutism was reported as a side effect [30]. The mechanism for the hair growth effect has not been accurately identified, but hair growth was reported to be induced by the increased nutritional supply due to vasodilation [31]. However, recently the media revealed that minoxidil was effective in suppressing male pattern hair loss by suppressing DHT production, and accordingly, more aggressive marketing promotions from related pharmaceutical companies came as a result. Minoxidil is reported to be the most effective treatment for initial hair loss, in which the hair partially falls out, and ongoing hair loss in the crown. So, only when it is used in the early stages of male pattern hair loss, can it prevent the progression of hair loss and contribute to hair growth. However, reportedly, it has no effect on the front of a scalp that has become bald, and if it is not applied to the scalp consistently twice a day for 6 to 12 months, no effect is seen [32].

In an experiment in which minoxidil was applied to the hair loss area, hair growth was promoted not only in men but also in women [33]. However, when 5% minoxidil was used, the color of the cilia became darker and thicker, resulting in hirsutism [34]. Therefore, in Korea, products for women are manufactured and sold at low concentrations. The side effects include salt and moisture retention; swelling; tachycardia; local peeling; dermatitis; skin irritation such as soreness; erythema; peeling at the site of administration [35]; itching; and dry skin due to a small amount of drug absorbed by the skin. Diffuse facial, forearm, cheek and neck hypertrichosis, redness, and contact dermatitis have been reported, and hirsutism can be induced in 0.5–1% of the users [36].

There may be a phenomenon in which the hair loss becomes worse by an increase in telogen hair shedding due to the temporary effect of minoxidil on the hair cycle [35]. Minoxidil’s hair growth-promoting effect depends on whether or not hair growth is initiated, and there is the disadvantage that hair returns to the state before treatment when minoxidil treatment is stopped [37]. Due to concerns about these side effects, in recent years, the social demand for the development of new anti-hair loss agents derived from natural products, whose efficacy is scientifically confirmed while being safe for the human body, has increased.

**Figure 4:**
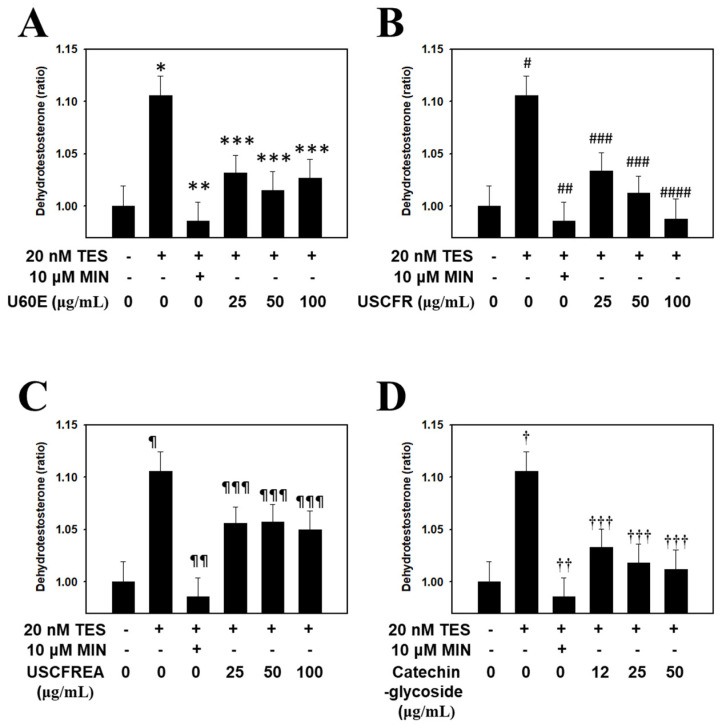
Inhibitory effect of *Ulmus* extract fractions on DHT production. Cells were pre−incubated with the indicated doses of (**A**) U60E, (**B**) USCFR, (**C**) USCFREA, and (**D**) catechin−glycoside (μg/mL, *w*/*v*) and treated with testosterone for 12 h. The DHT content (pg/mL) in cell lysates was measured according to the manufacturer’s instruction. Data are expressed as the mean ± SD. * *p* < 0.05, ^#^ *p* < 0.05, ^¶^ *p* < 0.05 and ^†^ *p* < 0.05 vs. normal group (blank). ** *p* < 0.005, *** *p* < 0.01, ^##^ *p* < 0.005, ^###^ *p* < 0.01, ^####^ *p* < 0.001, ^¶¶^ *p* < 0.005, ^¶¶¶^ *p* < 0.05, ^††^ *p* < 0.005 and ^†††^ *p* < 0.01 vs. control group (T, testosterone treated only). MIN—minoxidil, TES—testosterone.

Accordingly, in this study, experiments on the inhibition of DHT production were conducted with fractions of *Ulmus* extracts and catechin-glycoside, a single-compound isolated therefrom. The results were statistically significant compared to the positive and negative controls. The DHT reduction effect of U60E (Figure 4A), USCFR (Figure 4B), USCFREA (Figure 4C), and catechin-glycoside (Figure 4D) was confirmed to be effective for anti-hair loss and hair growth and suggests new agents for male pattern hair loss.

**Figure 5:**
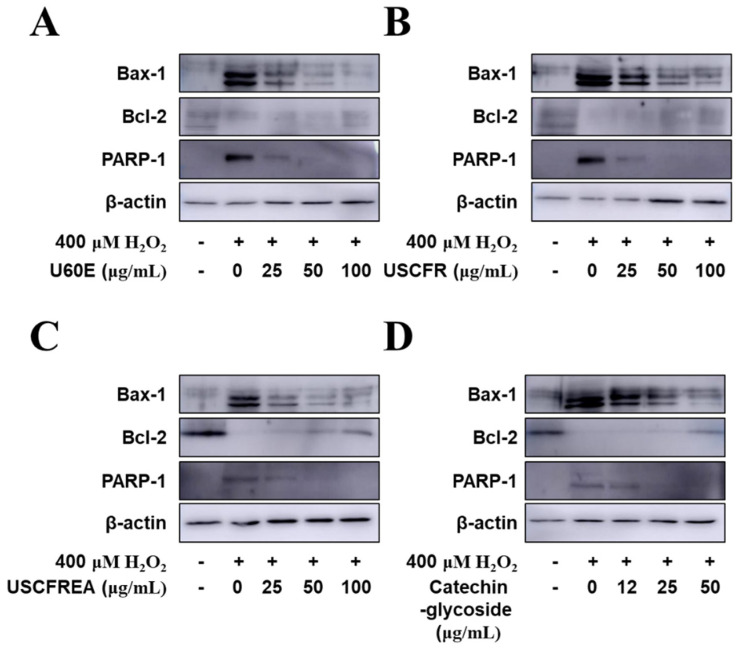
Anti−apoptotic effects of fractions of *Ulmus* extracts on H_2_O_2_−induced oxidative damage in HDFDC. Cells (1 × 10^5^ cells) were pre-incubated with (**A**) U60E, (**B**) USCFR, (**C**) USCFREA, and (**D**) catechin−glycoside (μg/mL, *w*/*v*) and further incubated with H_2_O_2_. Western blotting was performed to analyze levels of Bax−1, Bcl−2, PARP−1, and β−actin, as described in Materials and Methods. Data are expressed as the representative results from three independent experiments. ‘+’ and ‘−’ indicate present and absent, respectively.

Finally, we examined apoptotic signaling in response to H_2_O_2_ and the protective effects of *Ulmus* extract fractions in HDFPC cells (Figure 5). The administration of H_2_O_2_ increased the protein expression of apoptosis markers such as Bax and PARP, whereas H_2_O_2_ reduced Bcl-2 expression, an anti-apoptotic protein. The pretreatment with U60E (Figure 5A), USCFR (Figure 5B), USCFREA (Figure 5C), and catechin-glycoside (Figure 5D) markedly reduced the Bax and PARP protein expression levels. In contrast, Bcl-2 expression levels were markedly reduced in a dose-dependent manner. 

In previous reports, we determined cell viabilities in HFDPCs and other cells after challenges of oxidative stress or immunotoxic agents such as hydrogen peroxide [38] or lipopolysaccharides [39], respectively. Moreover, these effects were defined as multifunctional regulation through anti-oxidative, anti-inflammatory and/or anti-microbial pathways [39,40]. Particularly, oxidative-stress-induced programmed cell death, including apoptosis by hydrogen peroxide, was reproduced in ranges of 400~600 μM [38,40], and we examined the changes of apoptosis markers in HDFPCs. Moreover, U60E, USCFR, USCFREA and catechin-glycoside showed a relatively low toxicity in ~100 μM to HDFPCs (Figure 1A–D), suggesting that changes of oxidative-stress-induced TGF-β (Figure 2), IGF-1 level (Figure 3) and the TES-induced DHT production (Figure 4) were modulated in the non-toxic ranges of each materials. 

This study has two main limitations. First, the results of oxidative-stress-induced apoptosis and the increases in testosterone-induced DHT expression may differ from actual mechanisms of hair growth and loss. Nevertheless, the usages of these materials in life-care products, as opposed to medicinal drugs, specifically need to target such perceptions. Second, results may depend on the extraction methods of included raw materials; possible unpublished reports had different characteristics within certain facilities. For example, given that the extracts were in a complex, multifaceted condition for both substances and contents, it is not surprising that problems with efficacy attention processes emerged relatively frequently. This may be due to under-detection in the original studies or such factors simply being less significant, but this is first trial that used the remaining materials from supercritical fluid extracts.

These materials may be more applicable to usage than medicinal resource or/and natural constraints. In the future, more detailed experimental studies might improve the understanding of other molecular pathways activated by U60E, USCFR, USCFREA, and catechin-glycoside in various disease-related signaling processes and help guide prospective clinical studies evaluating their effects and appropriate usage.

## 3. Materials and Methods

### 3.1. Extraction of the Supercritical Fluid Extraction-Residues Extract of Ulmus davidiana

In this study, the *Ulmus davidiana* branch (with bark) extraction equipment was used for supercritical fluid extraction research. A *Ulmus davidiana* branch (with bark) was purchased from Yangnyeongsi Medicine Market (Seoul, Korea), and the impurities were removed, cleaned, and stored in dark room for use as experimental material. *Ulmus davidiana* branch with bark (100 kg) was extracted once with 60% edible ethanol at room temperature. Concentration was carried out by removing the ethanol under vacuum to afford 4.62 kg of extracted product (U60E). The dried sample was pulverized by passing through a pulverization net of 200 mesh, and maintained at 50 °C by controlling the temperature of the pulverization tank. When the temperature stabilized, the *Ulmus davidiana* branch (with barks) samples were kept in CO_2_ gas was maintained at the equilibrium pressure. Then, the pressure was controlled via the high-pressure pump until an experimental pressure of 400 bars was reached. After reaching the set pressure, extraction was performed by injecting a total of 100 L of ethanol (300 mL/min) over 333 min to the bottom of the extraction tank. The temperature and pressure of the high-pressure pump were maintained for 30 min to remove the residual ethanol in the sample, and the extraction was completed by flowing CO_2_ gas (NanoBio Research Center, Jeonnam Bioindustry Foundation, Jangsung, Jeonnam, Korea). As described above, the remaining materials of the extract of *Ulmus davidiana* branch (with barks) after supercritical extraction [12,14] were further extracted with 60% alcohol at room temperature and filtered. The extract was concentrated under vacuum concentration and freeze-dried to obtain 4.81 kg of the final product (USCFR) [14]. The filtrate (USCFR 1 kg) was fractionated with ethyl acetate, and the ethyl acetate layer extract was concentrated under vacuum and freeze-dried to obtain 185.2 g of the final product (USCFREA). USCFREA (185 g) was dissolved in water, and this aqueous solution was filtered through No. 20 filter paper (Hyundai Micro, Seoul, South Korea). Purification and isolation were performed by liquid column chromatography methods with thin layer chromatography monitoring. USCFREA (185 g) on a Disogel column (300 g, 3 × 50 cm) with a 30% methanol isocratic gradient in a Prep-LC system (20 mL/min, 280 nm) resulted in catechin 7-*O*-β-d-apiofuranoside (Compound **1**).

### 3.2. Cell Culture 

Primary HDFPCs and the appropriate media were obtained from the American Type Culture Collection (ATCC, Manassas, VA, USA). All cells were cultured at 37 °C in a humidified atmosphere with 5% CO_2_ and different complete media supplemented with 1% antibiotics and growth factors (ATCC). After at least 14 days of proliferation, the cells were used for in vitro experiments. 

### 3.3. In Vitro Experiments and Enzyme-Linked Immunosorbent Assay 

To investigate whether there are significant effects of U60E, USCFR, USCFREA and catechin-glycoside on oxidative-stress-induced cytokine production in HDFPCs, cells were pre-incubated with the indicated doses of each materials and treated with H_2_O_2_ for 12 h. To perform a test the protective effects of U60E, USCFR, USCFREA and catechin-glycoside on elevation of testosterone-induced DHT production in HDFPCs, cells were pre-incubated with the indicated doses of each material and treated with testosterone for 12 h. Results were compared with minoxidil (positive control) and negative control. Enzyme-linked immunosorbent assays (ELISA) were performed for IGF-I (ELH-IGF1-1, RayBiotech, Peachtree Corners, GA, USA), TGF-β (ADI-900-155, Enzo Biochem, New York, NY, USA) and dihydrotestosterone (DHT)(11-DHTHU-E01, ALPCO, Salem, NH, USA) The sample collection and assay protocols were adapted from the manufacturer’s instructions.

### 3.4. Western Blot Analysis

Cell protein extracts were prepared fresh before use as follows: cells (1 × 10^5^ cells) were pre-incubated with (A) U60E, (B) USCFR, (C) USCFREA, and (D) catechin-glycoside (μg/mL, *w*/*v*) at various concentrations or in control media for 10 min at 37 °C in a CO_2_ incubator. Then, the cells were treated with H_2_O_2_ (final concentration, 400 μM) and further incubated for 12 h. Then, harvested cells were solubilized by mixing with ice-cold lysis buffer (20 mM Hepes (pH 7.2), 1% Triton X-100, 10% glycerol, 1 mM EDTA, 1 mM phenylmethylsulfonyl fluoride, 50 mM NaF, 1 mM Na_3_VO_4_, leupeptin (10 mg/mL), pepstatin (10 mg/mL), and aprotinin (10 mg/mL)) by repeated trituration with a micropipette. The samples were then incubated for 1 h at 4 °C. Supernatants were obtained after centrifugation at 20,000× *g* for 10 min. The concentration of the extracted proteins in the supernatant was determined by the Bradford assay with bovine serum albumin (BSA) as a protein standard. Proteins at equivalent micrograms per lane were resolved by 7–12% sodium dodecyl sulfate-polyacrylamide gel electrophoresis (SDS-PAGE) and electrotransferred to polyvinylidene difluoride membranes (GE Healthcare, Brookfield, WI, USA) [41]. Antibodies against Bax, Bcl-2, PARP, and β-actin were used. Horseradish peroxidase-conjugated secondary antibodies (Santa Cruz Biotechnology, Santa Cruz, CA, USA) were used and the reactions were visualized by enhanced chemiluminescence. All immunoreactive signals were analyzed by densitometric scanning (LAS500, GE Healthcare, Brookfield, WI, USA).

### 3.5. Statistical Analysis

All data are expressed as the mean ± S.E.M. A one-way analysis of variance (ANOVA) followed by Tukey’s multiple range test was used to compare each group. Student’s t-test was used for comparisons between groups. Statistical analyses were conducted using SPSS for Windows software (ver. 10.0, Chicago, IL, USA). Data shown with different superscript letters are significantly different at *p* < 0.05.

## 4. Conclusions

This study was conducted to examine the anti-hair loss mechanism of the supercritical fluid extraction-residues extract of *Ulmus davidiana* by the regulation of cytokine productions and hormone function in human dermal follicle papilla cells. We investigated the modulatory effects on H_2_O_2_-induced cytokines, dihydrotestosterone hormone production, and anti-apoptosis effects. These results suggest that the supercritical fluid extraction-residues extracts of *Ulmus davidiana* contained functional molecules and were useful candidates for treating alopecia.

## Data Availability

Not applicable.

## Sample Availability

Samples of the compounds are available from the corresponding authors.
